# Cell-To-Cell Communication in Bilateral Macronodular Adrenal Hyperplasia Causing Hypercortisolism

**DOI:** 10.3389/fendo.2015.00034

**Published:** 2015-04-20

**Authors:** Hervé Lefebvre, Céline Duparc, Gaëtan Prévost, Jérôme Bertherat, Estelle Louiset

**Affiliations:** ^1^INSERM Unité 982, Laboratory of Neuronal and Neuroendocrine Differentiation and Communication, Mont-Saint-Aignan, France; ^2^Institute for Research and Innovation in Biomedicine, Rouen University, Mont-Saint-Aignan, France; ^3^Department of Endocrinology, Diabetes and Metabolic Diseases, University Hospital of Rouen, Rouen, France; ^4^INSERM Unité 1016, Institut Cochin, Paris, France; ^5^Department of Endocrinology and Metabolic Diseases, Hôpital Cochin, Assistance Publique-Hôpitaux de Paris, Paris, France

**Keywords:** Cushing’s syndrome, catecholamine, serotonin, ACTH, vasopressin, endothelin, leptin, illegitimate receptor

## Abstract

It has been well established that, in the human adrenal gland, cortisol secretion is not only controlled by circulating corticotropin but is also influenced by a wide variety of bioactive signals, including conventional neurotransmitters and neuropeptides, released within the cortex by various cell types such as chromaffin cells, neurons, cells of the immune system, adipocytes, and endothelial cells. These different types of cells are present in bilateral macronodular adrenal hyperplasia (BMAH), a rare etiology of primary adrenal Cushing’s syndrome, where they appear intermingled with adrenocortical cells in the hyperplastic cortex. In addition, the genetic events, which cause the disease, favor abnormal adrenal differentiation that results in illicit expression of paracrine regulatory factors and their receptors in adrenocortical cells. All these defects constitute the molecular basis for aberrant autocrine/paracrine regulatory mechanisms, which are likely to play a role in the pathophysiology of BMAH-associated hypercortisolism. The present review summarizes the current knowledge on this topic as well as the therapeutic perspectives offered by this new pathophysiological concept.

## Introduction

Chronic hypercortisolism results in a series of symptoms, including central obesity, skin changes, and arterial hypertension, known as Cushing’s syndrome. In 15–20% of cases, Cushing’s syndrome is the consequence of primary adrenal cortisol hypersecretion by bilateral adrenal hyperplasias or unilateral adrenocortical tumors. Bilateral macronodular adrenal hyperplasia (BMAH) is a rare cause of primary adrenal hypercortisolism representing <1% of all cases of Cushing’s syndrome (1). In this condition, cortisol hypersecretion by the enlarged adrenal glands leads to suppression of pituitary ACTH secretion. Consequently, the disease has long been named ACTH-independent macronodular adrenal hyperplasia (AIMAH). BMAH appears to be more frequent in women and hypercortisolism is usually diagnosed during the fifth and sixth decades (2, 3). In most patients with BMAH, hypercortisolism is moderate, contrasting with the important adrenal hypertrophy. The great majority of the published cases are sporadic but familial cases of the disease have also been reported (4). It should also be noticed that the extensive use of abdominal imaging, including computerized tomography (CT) scan and magnetic resonance imaging (MRI), has led to a marked increase in incidentally discovered BMAH (5). In this situation, BMAH is frequently associated with subclinical hypercortisolism (6).

At pathological examination, BMAH is characterized by an important increase in adrenal mass, which can reach 10–100 times the normal weight of the glands (7). The adrenal cortex is disorganized by the presence of large lipid-rich macronodules (8). There is no internodular atrophy and the nodules are usually not pigmented (9). At the microscopic level, the macronodules appear to be composed of two types of steroidogenic cells, i.e., large lipid-loaded cells, which are called spongiocytes, and small compact cells (7). Interestingly, these cell types display marked differences in steroidogenic enzyme expression. In fact, 17-hydroxylase is primarily detected in compact cells whereas 3β-hydroxysteroid dehydrogenase is principally expressed by spongiocyte cells (7, 10). This unequal repartition of steroidogenic enzymes among adrenocortical cells may result in relatively inefficient steroidogenesis, likely explaining the discrepancy between the major enlargement of the adrenal glands and the moderate intensity of hypercortisolism generally observed in patients with BMAH.

The pathophysiology of BMAH has long remained unknown. The bilaterality of the adrenal lesions suggested the occurrence of a pathogenic event affecting adrenal gland development during early embryogenesis. In fact, it is now known that BMAH is a genetically determined disease. Various mutational events can favor the development of the disease. The affected genes include the multiple endocrine neoplasia type 1 (*MEN1*), familial adenomatous polyposis (*APC*), phosphodiesterase 11A (*PDE11A*), G-protein αS subunit (*GNAS*), melanocortin type 2 receptor (*MC2R*), fumarate hydratase (*FH*), type A endothelin receptor (*EDNRA*), and protein kinase A catalytic subunit alpha (*PRKACA*) genes (6, 11–13). More recently, it has been shown that more than 50% of patients with BMAH carry mutations of the *ARMC5* gene, which behave as a tumor suppressor gene in the adrenal glands (14). In addition, *ARMC5* mutations may promote the development of a new multiple neoplasia syndrome associating BMAH and meningiomas (15).

The mechanisms involved in the pathogenesis of BMAH-associated cortisol hypersecretion are also better understood. It is indeed well established that, in BMAH tissues, cortisol secretion is stimulated by abnormally expressed membrane receptors, called illicit or illegitimate receptors, which supply the absence of pituitary ACTH (16). Several of these receptors are activated by circulating hormones, such as glucose-dependent insulinotropic peptide (GIP), luteinizing hormone (LH), and glucagon, while others bind paracrine regulatory signals released in the adrenal gland (16–21). More recently, it has been shown that, in addition to membrane G-protein-coupled receptors, BMAH tissues can abnormally express paracrine factors leading to formation of abnormal intraadrenal stimulatory loops, which seem to play an important role in cortisol hypersecretion (22–24). These illicit regulatory processes, which can be regarded as a pathological amplification of the paracrine systems physiologically occurring in the normal adrenal gland. In fact, it has been well established that the secretory activity of the normal adrenal cortex is influenced by various bioactive signals released in the vicinity of adrenocortical cells by chromaffin cells, neurons, cells of the immune system, adipocytes, and endothelial cells (25–27). The present review summarizes the current knowledge on the paracrine regulation of cortisol secretion in BMAHs from which emerges the new pathophysiological concept of paracrinopathy.

## Serotonergic Pathways in BMAH

In the normal adrenal gland, serotonin (5-hydroxytryptamine, 5-HT) is produced by perivascular mast cells (MC), which are primarily located in the subcapsular region of the cortex (28). The regulation of 5-HT release in the adrenal tissue is unknown but it is possible that 5-HT may be secreted in response to activation of the sympathetic system since adrenal MC have been shown to establish connections with cortical nerve endings (29). After its release, 5-HT is able to stimulate corticosteroid secretion through activation of 5-HT_4_ receptors positively coupled to adenylyl cyclase and calcium influx (28, 30, 31). It is not excluded that 5-HT may also influence corticosteroidogenesis through indirect mechanisms such as modulation of adrenal blood flow and/or production of cytokines by adrenocortical cells, as observed in rat (32, 33). *In vitro* studies have shown that adrenal 5-HT efficiently stimulates aldosterone secretion but only weakly activates cortisol production (31, 34). These differential actions on mineralo- and glucocorticoid synthesis likely result from the following observations: 5-HT is released by MC in the immediate vicinity of aldosterone-producing cells, and the 5-HT_4_ receptor is intensely expressed in zona glomerulosa but much more modestly in zona fasciculata (35, 36). In addition to its effect on the secretory activity of adrenocortical cells, adrenal 5-HT can be locally metabolized into inactive compounds such as 5-hydroxyindolacetic acid and 5-hydroxytryptophol (28, 34). This catabolic process is catalyzed by monoamine oxidase type A, which is mainly expressed by chromaffin cells (34).

In agreement with the data obtained *in vitro*, clinical studies have shown that administration of 5-HT_4_ receptor agonists, like zacopride and cisapride, to healthy volunteers induces a significant increase in plasma aldosterone levels without affecting plasma cortisol concentrations (30, 37–40). Interestingly, the stimulatory action of cisapride on aldosterone secretion was found to be additive with that of angiotensin II (38).

The physiological role of the serotonergic control of corticosteroid production remains unknown. However, several studies have shown that BMAH tissues exhibit several alterations in the adrenal serotonergic pathway, which tend to reinforce its stimulatory action on cortisol secretion. First, whereas MC represent the unique source of 5-HT in the normal adrenal, immunohistochemical studies have shown abnormal synthesis of 5-HT in a subpopulation of steroidogenic cells (22). Second, in some patients with BMAH, administration of the 5-HT_4_ receptor agonists, cisapride and metoclopramide, is followed by an abnormal elevation of plasma cortisol levels, suggesting an increased sensitivity of the adrenal hyperplastic tissue to 5-HT and 5-HT_4_ receptor agonists (19, 22, 41–44). In agreement with this hypothesis, *in vitro* studies conducted on tissue explants derived from BMAH previously responsive *in vivo* to 5-HT_4_ receptor agonists showed an increased potency and/or efficacy of 5-HT to stimulate cortisol production, in comparison with normal adrenal samples (22). Collectively, these data suggest that 5-HT exerts an intraadrenal stimulatory tone to stimulate cortisol secretion and is thus involved in the pathogenesis of BMAH-associated hypercortisolism. Consistently, 5-HT_4_ receptor antagonists were able to decrease cortisol secretion from perifused BMAH explants (36). Surprisingly, in some BMAH tissues, 5-HT was found to paradoxically inhibit cortisol secretion (45). This unexpected effect, which may counteract the influence of other stimulatory signals and may thus be beneficial by limiting the amplitude of cortisol hypersecretion, could result from abnormal coupling of eutopic 5-HT_4_ receptors to transduction pathways or illicit expression of 5-HT receptors negatively coupled to adenylyl cyclase such as the 5-HT_1_ and 5-HT_5_ types (46).

Clinical studies, by showing illicit cortisol responses to 5-HT_4_ receptor agonists in patients with BMAH, indicated that the effect of 5-HT on hyperplastic tissues was, at least in part, mediated by the eutopic 5-HT_4_ receptor. As expected, several groups reported an overexpression of the 5-HT_4_ receptor mRNA in BMAH tissues (42, 44, 47). Interestingly, the expression profile of 5-HT_4_ mRNA splicing variants seems to be different in BMAH samples from that observed in the normal adrenal (42). Immunohistochemical studies showed an ectopic distribution of the 5-HT_4_ receptor, which was visualized with high intensity in groups of cells localized in hyperplastic macronodules of the zona fasciculata (36). This result was consistent with the abnormal response of cortisol to 5-HT and 5-HT_4_ receptor agonists observed both *in vivo* and *in vitro*. However, in some BMAH tissues, the stimulatory effect of 5-HT on cortisol production was not modified by 5-HT_4_ receptor antagonists, indicating that the corticotropic action of the indolamine was mediated by other receptor types. Consistently, 5-HT was found to exert its biological effect on these tissues through activation of the 5-HT_7_ receptor (48). 5-HT_7_ receptor immunoreactivity could be visualized at the plasma membrane of adrenocortical cells throughout BMAH tissues, at variance with the normal adrenal gland in which the 5-HT_7_ receptor is exclusively detected in artery walls (48). Transcriptomic analyses have also shown an overexpression of the 5-HT_2B_ receptor in BMAH (49). However, the pathophysiological significance of this observation remains unclear since it is not known whether the 5-HT_2B_ receptor is expressed in adrenocortical cells or in blood vessels, as shown in various tissues (50).

In physiological conditions, 5-HT activates glucocorticoid synthesis through activation of the cAMP/PKA pathway (28, 51, 52). As expected, the stimulatory action of 5-HT on cortisol secretion by BMAH tissues was found to be suppressed by the PKA inhibitor H89 (48). These data are consistent with the observation that both the eutopic 5-HT_4_ receptor and the ectopic 5-HT_7_ receptor, which mediate the corticotropic effect of 5-HT in BMAHs, are positively coupled with adenylyl cyclase (46). However, the influence of 5-HT on steroidogenic enzyme expression in BMAH tissues remains currently unknown.

To summarize, in comparison with the normal adrenal gland, BMAH display molecular and cellular defects, which tend to reinforce the stimulatory effect of the intraadrenal serotonergic tone on cortisol production. These pathological findings include illicit synthesis of 5-HT in adrenocortical cells and aberrant expression of the 5-HT_4_ and 5-HT_7_ receptors. It thus appears likely that the enhancement of 5-HT paracrine pathways in BMAH tissues is involved in cortisol hypersecretion.

## Intraadrenal Production of ACTH

It has been shown in several mammalian species including man, that adrenomedullary chromaffin cells stimulate the secretory activity of adrenocortical cells through a paracrine mode of communication involving diverse bioactive signals (53). In particular, it has been shown that chromaffin cells are able to express the gene encoding the precursor of ACTH proopiomelanocortin (POMC) and to synthesize detectable amounts of ACTH (54, 55). The presence of chromaffin ACTH-producing cells has been observed in BMAH tissues as early as 2001 (56). A few years later, several groups reported illicit expression of POMC and synthesis of ACTH in adrenocortical cells in isolated cases of BMAH (57–60). More recently, the presence and role of ACTH was systematically investigated in a large series of 30 cases of BMAH (24). The tissues were found to express POMC mRNA at variable levels. The presence of proconvertase 1, a protease involved in the processing of POMC into ACTH, was also detected in a subpopulation of adrenal cells suggesting that POMC could be converted into ACTH in the hyperplastic tissues. In fact, immunohistochemical studies revealed the presence of ACTH immunoreactivity in chromaffin cells of the adrenal medulla and, as previously noticed, in some adrenocortical cells either isolated or arranged in small clusters disseminated in the tissues. Adrenocortical ACTH-positive cells exhibit the usual characteristics of steroidogenic cells, i.e., loaded with numerous lipid inclusions, and express several markers of steroidogenic differentiation including steroidogenic factor 1 (SF1), the HDL-cholesterol receptor SRB1 (scavenger receptor B1), and 17-hydroxylase. Thus, they represent a subcategory of adrenocortical steroidogenic cells that abnormally express ACTH. The ectopic synthesis of ACTH in these cells is not the consequence of abnormal corticotropic-like differentiation as indicated by the lack of significant T-pit [a transduction factor which drives pituitary corticotrophs differentiation (61)] expression in the tissues (24). The presence of ACTH in adrenocortical cells may rather be regarded as an additional trait of the previously reported neuroendocrine differentiation of the hyperplastic tissues (21, 22, 48). Interestingly, ACTH-positive cells were also labeled by antibodies directed against the Leydig cell marker insulin-like 3 (INSL3) indicating that ACTH synthesis may result from illicit gonadal-like differentiation of some adrenocortical cells (24). This observation is consistent with the data obtained from older studies showing that testicular Leydig cells and ovarian granulosa cells are able to express POMC and synthesize ACTH (62, 63). The expression of gonadal markers in the adrenal hyperplastic tissues is also reliable with previous reports of BMAH-associated with androgens or estrogens overproduction (8, 64–66). As the adrenal glands and gonads derive from a same tissue precursor, the adrenogonadal primordium, it is likely that the presence of gonadal-like cells in the adrenal tissues may result from abnormal differentiation and/or separation of the adrenogonadal primordium during early embryogenesis explaining the bilaterality of the lesions.

**Figure 1:**
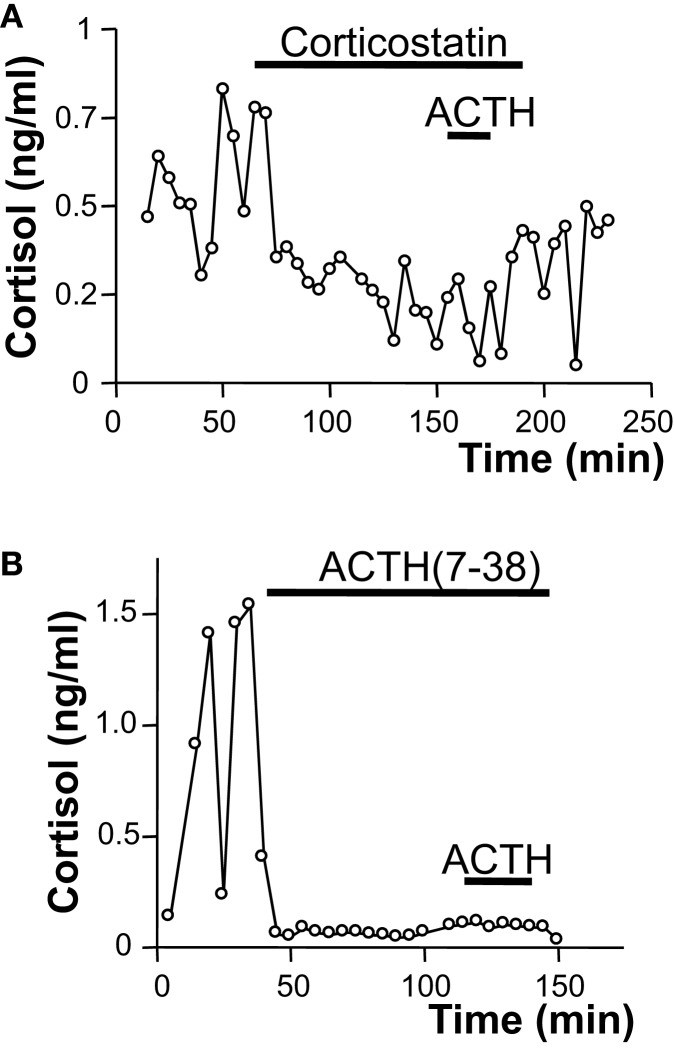
**Role of intraadrenal ACTH secretion in the control of cortisol production by bilateral macronodular adrenal hyperplasia (BMAH) associated with Cushing’s syndrome**. Spontaneous and ACTH-induced cortisol secretions by perifused BMAH explants were inhibited by application of the melanocortin type 2 receptor (MC2R) antagonists corticostatin [2 × 10^−7^ M; **(A)**] and corticotropin (7–38) [10^−7^ M; **(B)**]. Corticostatin and corticotropin (7–38) significantly reduced the amplitude of cortisol pulses.

*In vitro* studies revealed that ACTH is released by BMAH tissues in a pulsatile way, consistently with former clinical studies showing a pulsatile mode of cortisol secretion in patients with BMAH (67). The ectopic secretion of ACTH by the adrenal glands could also be observed *in vivo* in two patients through adrenal vein catheterization (24). In fact, adrenal vein sampling demonstrated a significant ACTH concentration gradient between the adrenal versus peripheral veins as well as inferior petrosal sinus in one of the two patients (24, 36). All these results suggested that ACTH produced by intraadrenal gonadal-like cells may stimulate cortisol secretion in BMAH tissues, supplying therefore pituitary ACTH, which is suppressed by cortisol excess. This assumption could be assessed by the following observations: ACTH and cortisol levels were positively correlated in culture medium during perifusion of BMAH samples; basal plasma cortisol concentrations measured in the patients were positively correlated with both the levels of *POMC* mRNA and the ACTH histological score in the tissues; the ACTH receptor (MC2R) antagonists corticostatin and ACTH (7–38) significantly inhibited the production of cortisol *in vitro* by BMAH explants (24). Interestingly, MC2R antagonists also markedly reduced the amplitude of cortisol pulses indicating that oscillations in glucocorticoid production are determined by ACTH-secreting cells (Figure 1). Although globally underexpressed (47), MC2R was upregulated by ACTH in BMAH tissues, as previously established in the normal adrenal gland (68). MC2R mRNA levels were indeed positively correlated with POMC mRNA rates and MC2R immunoreactivity was primarily observed in the vicinity of ACTH-positive cells, which were also found to express the receptor (24). Thus, it seems that intraadrenal ACTH may exert autocrine actions in BMAH. The regulation of ACTH production by BMAHs has also been investigated by using the same *in vitro* approach. Dexamethasone and the glucocorticoid receptor antagonist RU486 failed to influence ACTH release indicating that, at variance with pituitary ACTH, intraadrenal ACTH is not regulated by cortisol (24). Conversely, it was observed that several ligands of illicit membrane receptors, i.e., 5-HT, LH/hCG, and GIP, stimulate ACTH release from BMAH explants by increasing pulse amplitude without affecting pulse frequency (24). This unexpected finding suggested that activation of membrane receptors may stimulate cortisol production via two mechanisms including a direct effect on corticosteroidogenesis, as previously shown in BMAH cell culture (22), and an indirect action via ACTH secretion (24). In agreement with this hypothesis, it was observed that MC2R antagonists reduce the amplitude of the cortisol response to GIP. It seems therefore that intraadrenal ACTH is a common intermediate and amplifier of the action of several illicit membrane receptors in BMAH tissues.

## Catecholaminergic Pathway in BMAH

**Figure 2:**
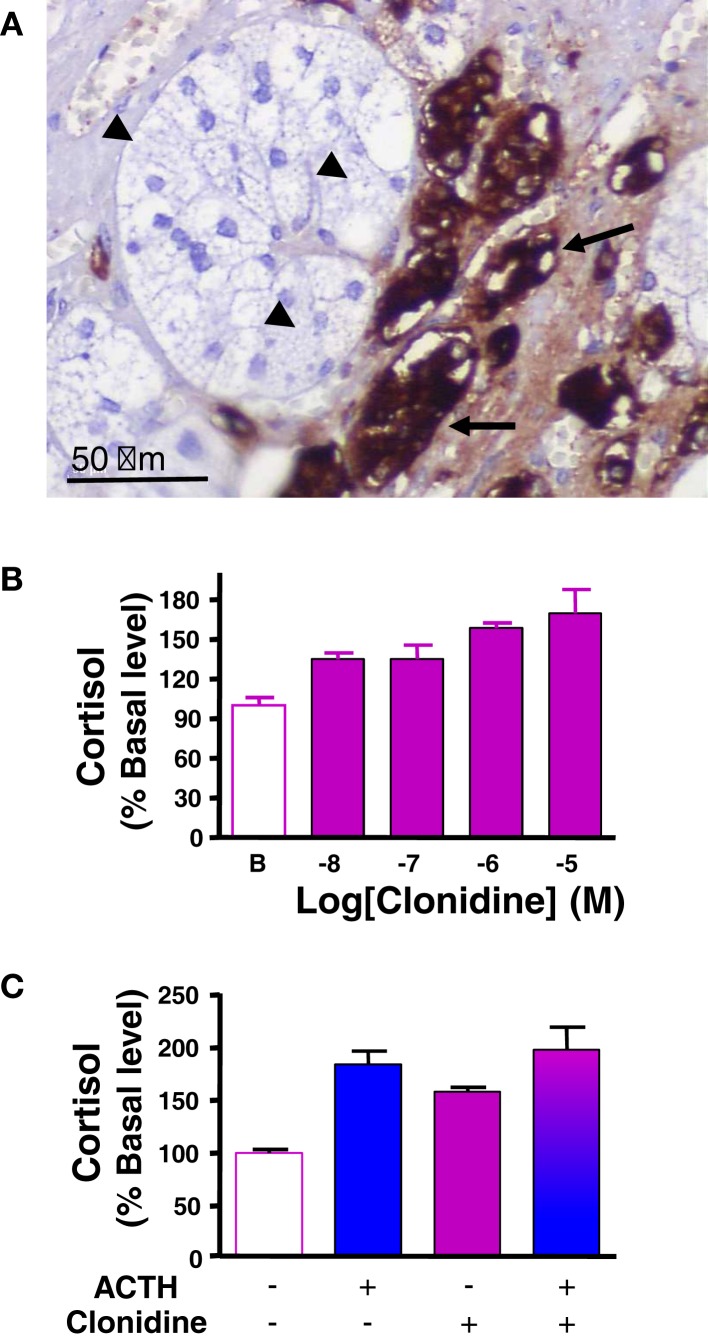
**Catecholaminergic pathway in bilateral macronodular adrenal hyperplasia (BMAH) associated with Cushing’s syndrome**. **(A)** Chromogranin A-positive chromaffin cells (arrows), which represent the main source of catecholamines in the normal adrenal gland, were in close contact with steroidogenic cells (arrow heads) in BMAH tissue. **(B)** Clonidine, an α2 receptor agonist, dose-dependently stimulated cortisol secretion by cultured adrenocortical cells derived from a BMAH tissue. Adapted from Ref. (47). **(C)** The maximum cortisol responses of cultured BMAH adrenocortical cells to high concentrations of ACTH (10^−10^ M) and clonidine (10^−6^ M) were not additive, suggesting that α2 and MC2R receptors are coupled to a common transduction pathway.

The catecholamines adrenaline and noradrenaline are secreted by adrenal chromaffin cells under control of splanchnic nerve and proinflammatory cytokines. It has been hypothesized that catecholamines released by chromaffin cells present at the corticomedullary junction and in the cortex, may influence steroid production by adrenocortical cells, in particular during stress and inflammation (25, 27, 69). In support of this hypothesis, *in vitro* experiments have demonstrated that adrenaline and noradrenaline are able to modulate glucocorticoid production in frog and bovine adrenocortical cells (70, 71). However, there is no clear evidence for catecholamine responsiveness in human adrenal, since noradrenaline did not affect *in vitro* cortisol secretion by human normal adrenocortical cells (72). By contrast, abnormal catecholaminergic control of steroidogenesis has been documented in some patients with macronodular adrenal hyperplasia-associated with Cushing’s syndrome. Indeed, immunohistochemical studies have revealed the presence of clusters of chromogranin A-immunopositive chromaffin cells in the vicinity of steroidogenic cells, indicating paracrine interactions between the two cell types in hyperplastic tissues (24) (Figure 2A). In addition, abnormal elevations of plasma cortisol have been detected in patients placed in physiological conditions associated with increases in endogenous catecholamine, such as upright posture or insulin-induced hypoglycemia (22, 73). Moreover, increases in circulating cortisol levels provoked by administration of isoproterenol, a β-adrenergic receptor agonist, as well as decreases in plasma cortisol concentrations in response to infusion of propranolol, a β blocker, have given evidence for illicit β-adrenergic control of steroidogenesis (73–76). Aberrant expression of β adrenergic receptors in BMAH tissues has been confirmed by binding, RT-PCR, and functional *in vitro* experiments (22, 59, 73, 77). In particular, hypersensitivities to salbutamol and isoproterenol, two β2 receptor agonists, have been observed on cultured cells derived from BMAH tissues (22, 59). Our group has also demonstrated, by using molecular and cellular biological approaches, the occurrence of illegitimate α2-adrenergic receptors in BMAHs (47). In particular, *in vivo* and *in vitro* experiments have revealed that administration of the α2 receptor agonist clonidine stimulated cortisol synthesis in one BMAH case (47) (Figure 2B). Pharmacological studies have shown that the positive effect of clonidine on cortisol production resulted from activation of α2 receptors positively coupled to the adenylyl cyclase/PKA pathway (47). The absence of additive effects of high concentrations of ACTH and clonidine on cortisol production is consistent with a common transduction pathway for α2 and MC2R receptors (Figure 2C). Altogether, these data indicate that, in some BMAH tissues, the presence of chromaffin cells intermingled with steroidogenic cells expressing illegitimate β- or α2-adrenergic receptors, give rise to a positive adrenergic regulatory loop, which likely contributes to the pathogenesis of hypercortisolism.

## Vasopressinergic Pathway in BMAH

Arginine vasopressin (AVP) is known to activate glucocorticoid production through a dual action on the hypothalamic–pituitary–adrenal axis. AVP released by hypothalamic neurons is a potent stimulator of ACTH production by pituitary corticotrophs via vasopressin type 1b (V_1b_) receptors (78, 79). In addition, AVP can be released by adrenomedullary chromaffin cells and act as a paracrine modulator of glucocorticoid production through activation of type 1a receptors (V_1a_) positively coupled to phospholipase C (78, 79). However, the physiological role of intraadrenal AVP is not known and *in vivo* administration of AVP or its analogs to dexamethasone-pretreated healthy volunteers has no influence on plasma cortisol levels (80, 81). Surprisingly, abnormal plasma cortisol responses to AVP have been observed in patients with BMAH-associated hypercortisolism. AVP-induced increase in cortisol levels was observed in response to injection of AVP analogs or hypertonic saline test, which increases endogenous AVP release, in the absence of any significant variation of plasma ACTH concentration (41, 80, 82). The enhanced sensitivity of adrenocortical cells to AVP has been confirmed *in vitro* by perifusion and cell culture experiments (22, 59, 82). RT-PCR and pharmacological studies have revealed that some BMAH tissues overexpress the eutopic V_1a_ receptor subtype (23, 83, 84) and/or abnormally synthesize ectopic V_1b_ and V_2_ receptors (23, 85, 86). Involvement of AVP and V_1a_ receptors in hypercortisolism has been confirmed in a patient with an AVP-sensitive BMAH in whom oral administration of a non-peptidic V_1a_ antagonist significantly decreased urinary cortisol level (82).

It is conceivable that circulating AVP may control cortisol secretion in patients with BMAH expressing illicit vasopressin receptors. However, basal plasma AVP levels (around 10^−12^ M) are much lower than the minimal effective dose of AVP (around 10^−10^ M) to stimulate cortisol release by BMAH tissues *in vitro* (23). It seems therefore more likely that illegitimate adrenal AVP receptors are predominantly activated by locally produced AVP through a paracrine mechanism similar to that observed in the normal adrenal gland. In this respect, BMAH tissues have been shown to contain two types of AVP producing cells, identified as chromaffin and steroidogenic cells, the latter clearly representing an ectopic source of the nonapeptide (22). Collectively, these data indicate that a vasopressinergic loop, resulting from aberrant intraadrenal AVP production and overexpression of functional V_1a_/V_2_ receptors, is involved in the pathophysiology of cortisol excess in some patients with BMAH.

## Other Paracrine Regulatory Mechanisms

**Figure 3:**
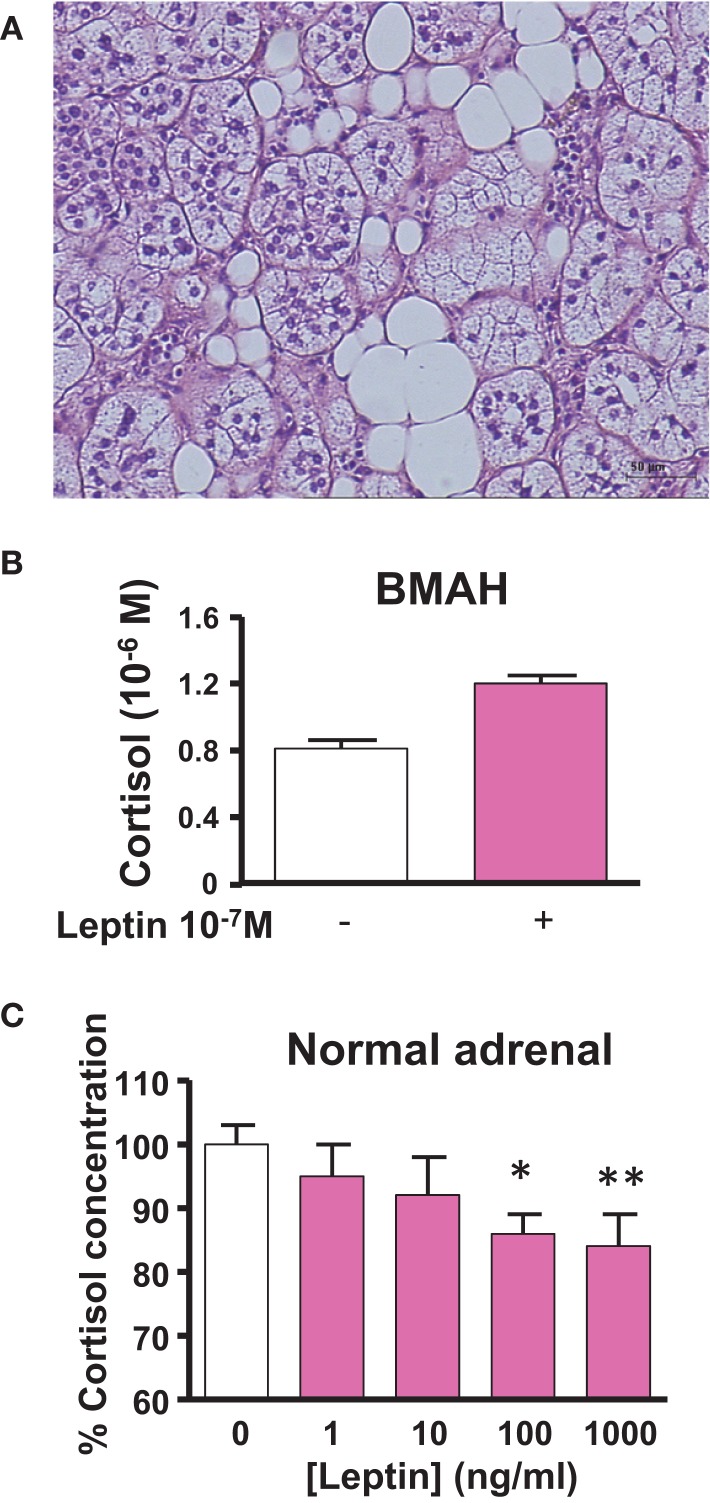
**Leptin pathway in bilateral macronodular adrenal hyperplasia (BMAH) associated with Cushing’s syndrome**. **(A)** Islets of adipocytes in the vicinity of steroidogenic cells in BMAH tissue. **(B)** Abnormal stimulatory effect of leptin (10^−7^ M) on cortisol secretion by cultured adrenocortical cells derived from a BMAH tissue in a patient with Cushing’s syndrome (*p* = 0.06). Adapted from Ref. (89). **(C)** Leptin reduces ACTH-induced cortisol secretion by cultured normal adrenocortical cells in a dose-dependent manner (*p < 0.01; **p < 0.001). Adapted from Ref. (87).

Like the kidneys, the adrenal gland is surrounded by adipose tissue, which may release numerous bioactive substances capable of influencing the secretory activity of steroidogenic cells. Among them, leptin has been shown to dose-dependently inhibit ACTH-induced cortisol secretion through activation of the leptin receptor and repression of *CYP17* expression in adrenocortical cells (87, 88). Thus, it seems that leptin produced by the periadrenal adipose tissue may act as a metabolic signal to exert a negative control on cortisol production. Interestingly, BMAH tissues have been shown to contain clusters of adipocytes sometimes arranged in lipomatous islets (Figure 3A), suggesting that the paracrine control of cortisol secretion by leptin could be reinforced in comparison with the normal adrenal gland (21). However, at variance with the physiological process, leptin was found to paradoxically stimulate cortisol release in some BMAH tissues and thus participate in the pathophysiology of hypercortisolism (89) (Figures 3B,C). This illicit cortisol response to leptin may result from abnormal coupling of leptin receptors to transduction pathways.

Finally, the adrenal cortex is a richly vascularized organ so that each adrenocortical cell is in close contact with at least one capillary (90). As expected, endothelin and the endothelin-converting enzyme were detected at both mRNA and protein levels in the adrenocortical tissue (91). In addition, adrenocortical cells were found to express the endothelin types A (ETA) and B (ETB) receptors and endothelin-1 is able to stimulate both aldosterone and cortisol production by normal adrenocortical cells (92, 93). Although several studies indicate that endothelin may play a role in the pathophysiology of aldosterone-secreting neoplasms, it is not known whether this peptide may be involved in BMAH-associated hypercortisolism. However, a mutation of the *EDNRA* gene, which encodes the ETA receptor, has been found in a familial case of BMAH suggesting that a defect in the adrenal endothelin pathway may favor the development of adrenal hyperplasia and hypercortisolism (12).

## Integrative Pathophysiology of BMAH-Associated Hypercortisolism

**Figure 4:**
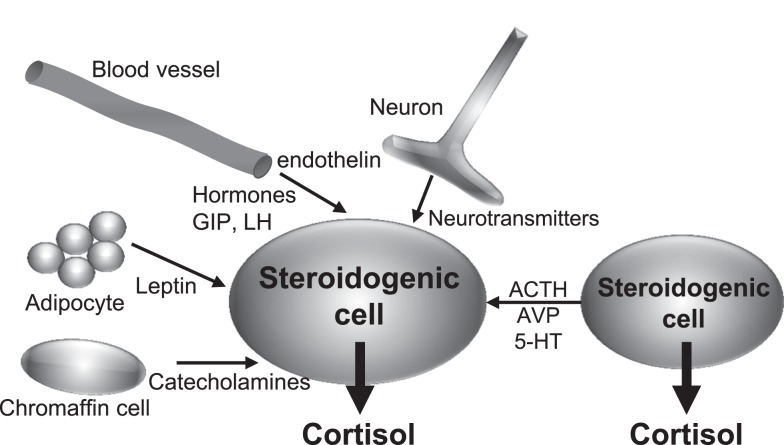
**Schematic representation of endocrine and paracrine controls of cortisol secretion in bilateral macronodular adrenal hyperplasia (BMAH) associated with Cushing’s syndrome**. AVP, vasopressin; 5-HT, serotonin; GIP, glucose-dependent insulinotropic peptide; LH, luteinizing hormone.

The studies recently published have brought important new insights into the comprehension of the pathophysiology of BMAH, which will undoubtedly stimulate the research on the disease and other adrenal disorders. In particular, it is now unquestionable that BMAH is a genetically determined condition, *ARMC5* being a major susceptibility gene of the disease. However, the mechanisms by which *ARMC5* favors the development of hyperplasia and hypercortisolism are still unknown. In particular, the pathophysiological processes linking *ARMC5* mutations and the initiation of illicit paracrine regulatory loops will have to be identified. However, all the data summarized in the present review suggest the following sequence of pathogenic events. First, it is likely that the causative mutations of the disease alter adrenal embryogenesis leading to the abnormal presence of gonadal-like cells in the adrenal areas. Progressive expression of POMC and ACTH by these cells then results in adrenocortical hyperplasia and hypercortisolism via activation of the cAMP/pKA pathway by the MC2R. Illicit expression of some membrane receptors may be regarded as a witness of the gonadal-like differentiation of the tissues. This is particularly the case for the LH, GIP, and 5-HT_7_ receptors, which are known to be physiologically expressed in the gonads (94, 95). On the other hand, it is conceivable that local production of ACTH may also result into overexpression of membrane receptors and their ligands. This hypothesis appears particularly relevant for the regulation of BMAH tissues by 5-HT. Indeed, an increase in 5-HT_4_ mRNA levels has been noticed in adrenal glands removed from patients with ACTH-dependent (Cushing’s disease) hypercortisolism in comparison with normal adrenals (47). Intraadrenal ACTH may also be responsible for the unusual expression pattern of 5-HT_4_ isoforms in BMAH tissues since recent studies have shown that ACTH globally alters mRNA splicing in adrenocortical cells (96). In addition, important insights have been provided by studies conducted on animal models. In rats, chronic stress, which stimulates ACTH release by the pituitary corticotrophs, induces a significant increase in the expression of the eutopic adrenal 5-HT receptor, which is the 5-HT_7_ receptor, as well as abnormal synthesis of 5-HT in clusters of adrenocortical cells (97). The illicit serotonergic loop observed in human BMAH tissues may therefore be regarded as an abnormal activation of a physiological mechanism, which is probably aimed at potentiating the glucocorticoid response to stress. This process may be driven by intraadrenal ACTH and subsequent activation of PKA, which can also be stimulated in BMAH tissues by somatic and/or germline mutations such as those affecting the *PDE11A* and *PRKACA* genes (13, 98) or cAMP-coupled illicit membrane receptors like the LH, GIP, and 5-HT_7_ receptors (16, 48). Collectively, these data suggest that intraadrenal paracrine regulatory loops may be regarded as valuable targets for new pharmacological treatments of BMAH-associated hypercortisolism (Figure 4). Especially, inhibition of the action of locally produced ACTH, which seems to represent a common intermediate to the influence of several types of abnormally expressed membrane receptors in BMAH tissues, may be a particularly efficient strategy. MC2R antagonists, which are currently under clinical development for the treatment of hypercortisolism associated to Cushing’s disease, will have thus to be evaluated in patients with primary adrenal Cushing’s syndrome due to BMAH.

## Conflict of Interest Statement

The authors declare that the research was conducted in the absence of any commercial or financial relationships that could be construed as a potential conflict of interest. The Guest Associate Editor Antoine Martinez declares that, despite having collaborated with author Jérôme Bertherat, the review process was handled objectively and no conflict of interest exists.
